# Perianal Sonographic Approach to Fistula-in-Ano and Perianal Abscesses: A Pictorial Review

**DOI:** 10.7759/cureus.103963

**Published:** 2026-02-20

**Authors:** Takahiro Hosokawa

**Affiliations:** 1 Radiology, Saitama Children's Medical Center, Saitama, JPN

**Keywords:** fistula, fistula-in-ano, perianal abscess, sonography, ultrasound

## Abstract

Fistula-in-ano and perianal abscesses are common conditions in pediatric patients. Recently, the perianal ultrasound approach has been reported as a useful method for evaluating anal and perianal lesions. However, this technique remains unfamiliar to many sonographers. In this pictorial review, we aimed to describe the basic sonographic approach for assessing fistula-in-ano and perianal abscesses. A sagittal view of the anal canal is obtained by placing the probe on the proctodeum parallel to the intergluteal cleft, providing a longitudinal image of the anus and rectum. The probe is then gently swung from right to left to identify anatomical structures and detect fistula-in-ano or perianal abscesses. When necessary, the probe is rotated perpendicular to the gluteal cleft to obtain additional transverses, offering further diagnostic information. An abscess appears as a hypoechoic (low-echoic) mass surrounded by echogenic subcutaneous tissue and a hypervascular rim due to inflammation. The presence of a fluid-filled, low-echoic mass indicates pus accumulation, prompting surgical incision. The fistula-in-ano typically extends from a rectal crypt to the dermis, and abscess formation may occur along this tract. Perianal ultrasonography is a valuable, noninvasive tool for evaluating perianal lesions, and sonographers should become familiar with this approach. Throughout the procedure, patient comfort and pain management are essential, and further diagnostic imaging or intervention may be warranted when necessary.

## Introduction and background

Fistula-in-ano and perianal abscesses are common in pediatric patients, with a reported incidence ranging from 0.5% to 4.3% [1-5], and are often associated with inflammatory diseases [2,3,6-8]. Magnetic resonance imaging (MRI) is considered the gold standard for evaluating these conditions [7-10]. Recent studies by pediatric physicians managing pediatric patients with inflammatory diseases have shown that the perianal ultrasound approach is a useful alternative for assessing anal and perianal lesions [1,2,6,11]. However, to our best knowledge, there were no reports focusing on the practical approach methods of perineal ultrasound.

Ultrasound offers advantages such as the absence of radiation exposure, the ability to perform bedside evaluations, and the possibility of repeated examinations without general anesthesia. Nevertheless, perianal ultrasonography remains unfamiliar to many sonographers, and detailed sonographic approaches for fistula-in-ano and perianal abscesses have not been thoroughly described in previous literature [2,6,11-14]. This knowledge gap makes it challenging for sonographers to perform initial assessments confidently.

In this pictorial technical narrative review, we aim to describe a sonographic technique for detecting fistula-in-ano and perianal abscesses, present representative images from pediatric cases, and propose imaging strategies for patient management.

## Review

Basic knowledge of fistula-in-ano and perianal abscess for patient evaluation

**Figure 1:**
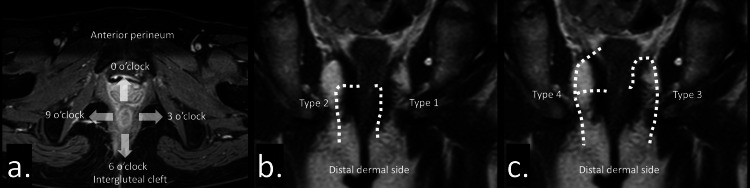
Anatomical location of the fistula. (a) The anal clock represents the anterior perineum, left edge, intergluteal cleft side, and right edge, which are represented as the 12, 3, 6, and 9 o’clock positions, respectively. (b) Intersphincteric (type 1) and transphincteric (type 2) fistulas. (c) Suprasphincteric (type 3) and extrasphincteric (type 4) fistulas.

Anatomical location of the fistula is important for determining the treatment approach (Figure 1a). The anal clock is useful for indicating fistula location. The anterior perineum, left edge, intergluteal cleft side, and right edge are represented as the 12, 3, 6, and 9 o’clock positions, respectively [15]. In addition, the relationship between the fistula tract, from the dentate line to the dermal opening, and the anal sphincter is important for determining the treatment plan, particularly the surgical approach [16]. The Parks classification categorizes fistulas into four types: type 1, intersphincteric; type 2, transphincteric; type 3, suprasphincteric; and type 4, extrasphincteric [17] (Figures 1b, 1c). To minimize anal sphincter injury, an appropriate treatment plan, including antibiotics, biologic therapy, and surgical interventions such as incision, drainage, fistulotomy, or seton placement, should be selected [16].

Sonographic approach for fistula-in-ano and perianal abscess

Basic sonographic procedure for fistula-in-ano and perianal abscess was followed [1,2,6,10,12,13,18].

**Figure 2:**
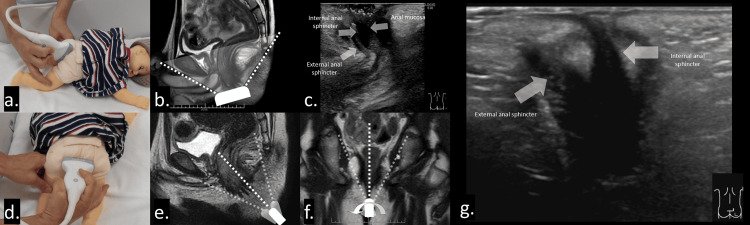
Approach for fistula-in-ano and perianal abscess. Patients are positioned in the left or right lateral decubitus position or in the prone position to facilitate optimal probe placement and visualization of the anal canal and surrounding tissues. (a, b) Sagittal view of the anal canal obtained by placing the probe on the proctodeum parallel to the intergluteal cleft, allowing longitudinal visualization of the anus and rectum. (c) The anal canal appears as concentric, alternating hypoechoic and hyperechoic layers, corresponding to the internal and external anal sphincters and the anal mucosa, respectively. (d) Probe placement perpendicular to the gluteal cleft. (e) When the probe is positioned perpendicular to the gluteal cleft, the distance between the proctodeum and the probe surface increases. Although a longitudinal view of the anus may be produced, it is difficult to maintain because small probe deviations can result in significant displacement of the imaging plane. (f) The probe is gently swung from right to left (or vice versa) to evaluate surrounding structures. (g) Coronal view of the anal canal obtained with the probe placed perpendicular to the gluteal cleft, demonstrating the low-echoic internal and external anal sphincters.

First, appropriate patient positioning is essential. The patient is placed in the left or right decubitus position or in the prone position; in some situations, the supine position may be adequate. Second, an appropriate probe (linear or convex) is selected based on the patient’s age and lesion depth. A high-frequency linear transducer (9-15 MHz) is generally suitable for evaluating subcutaneous lesions. For large abscesses or deeply located lesions, a convex transducer with a frequency range of 2-9 MHz may be more appropriate. Color or power Doppler techniques can also assist in assessing inflammatory activity. Third, to obtain the sagittal view, the probe is positioned on the proctodeum parallel to the intergluteal cleft (Figures 2a, 2b), allowing visualization of the anus and rectum in longitudinal section (Figure 2c). Positioning the probe perpendicular to the gluteal cleft (Figure 2d) is generally inadequate at this stage, as the increased distance between the probe and the proctodeum makes it difficult to maintain a stable longitudinal view. Even a slight probe deviation can result in significant displacement of the imaging plane (Figure 2e). Fourth, the sagittal view provides key anatomical information and helps identify the location of the anal canal. Fifth, the probe is gently swung from right to left (or vice versa) to evaluate the surrounding tissues (Figure 2f). Sixth, fistulas and abscesses are localized using the identified anatomical landmarks. Seventh, a transverse view may be obtained when additional information is required. The probe is rotated perpendicular to the gluteal cleft (Figure 2d) to acquire a transverse image, providing complementary visualization of the anal canal and perianal lesions (Figure 2g).

Imaging finding

Treatment of perianal abscesses and fistula-in-ano remains controversial; however, commonly used options include antibiotic therapy, abscess incision and drainage, and fistulectomy. Both conservative and surgical approaches have been reported to achieve favorable outcomes [19]. Therefore, the indication for surgical intervention should be determined based on each patient’s clinical condition, such as abscess progression or lack of symptomatic improvement during antibiotic therapy [3,20-23]. To guide appropriate treatment selection, the presence or absence of abscesses and fistulas, as well as abscess size, must be carefully evaluated. Ultrasonography can be used to visualize and assess these findings.

Fistula-in-ano

**Figure 3:**
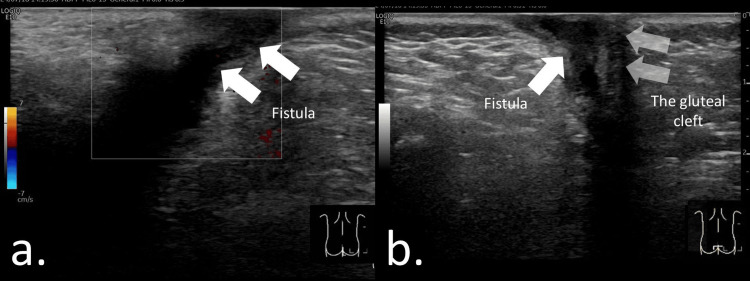
Fistula-in-ano at the 1 o’clock position in a nine-month-old boy. (a) A sagittal view of the anal canal was obtained by placing the probe parallel to the intergluteal cleft. A low-echoic linear structure consistent with a fistulous tract was visualized. No definite low-echoic fluid collection suggestive of an abscess was identified. Fistula-in-ano was diagnosed based on the sonographic findings. (b) A coronal view of the anal canal was obtained with the probe placed perpendicular to the gluteal cleft, demonstrating a low-echoic fistulous tract on the right side of the anal canal at the 1 o’clock position.

**Figure 4:**
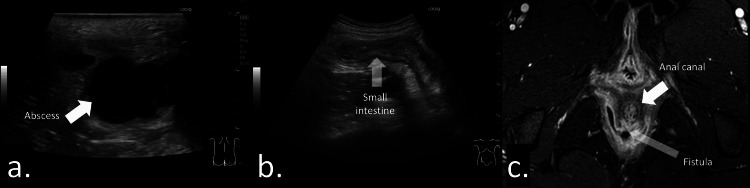
Inflammatory disease presenting with anal pain at the 12 o’clock position in a 15-year-old female. (a) Sonographic image demonstrating a large perianal abscess. The fistulous tract originating from the anal crypt could not be visualized because of the large lesion size. (b) Abdominal sonogram showing inflammatory changes in the terminal ileum. (c) Axial gadolinium-enhanced, fat-suppressed T1-weighted magnetic resonance image obtained after surgical incision, demonstrating a fistulous tract arising from the anal canal at the 6 o’clock position.

A fistula-in-ano typically extends from the rectal crypt to the dermis (Figure 3a) and may be associated with abscess formation along the tract [2,12]. The distal tract commonly connects the abscess to the dermis, whereas the proximal tract extends from the rectal crypt to the abscess cavity (Figures 3a, 3b). Visualization of the proximal portion can be challenging because of its deep location, large abscess size (Figure 4a), or spontaneous closure prior to imaging. In Video 1, a giant abscess is demonstrated, making it difficult to identify the fistulous connection to the abscess and to visualize the entire extent of the lesion.

**Video 1:** Video demonstrating an inflammatory disease presenting with anal pain at the 12 o’clock position in a 15-year-old female. Sagittal view of the anal canal obtained by placing the probe parallel to the intergluteal cleft. A large low-echoic abscess is visualized; however, the full extent of the lesion cannot be adequately assessed, and a fistulous tract is not identifiable. A diagnosis of perianal abscess was made, and a surgical incision was performed.

Perianal abscess

**Figure 5:**
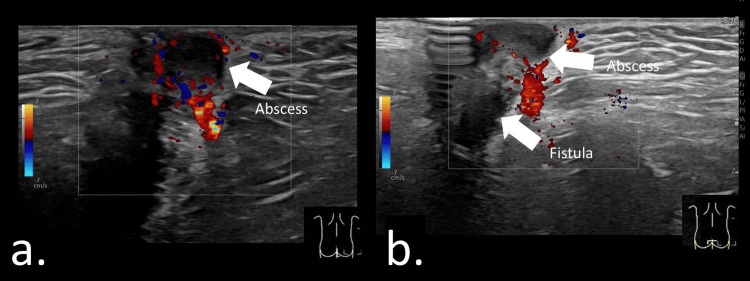
Perianal abscess and fistula-in-ano at the 11 o’clock position in a six-month-old boy. (a) A sagittal view of the anal canal was obtained by placing the probe parallel to the intergluteal cleft. A 10-mm hypoechoic fluid collection was identified. (b) A coronal view of the anal canal was obtained with the probe placed perpendicular to the gluteal cleft. A hypoechoic linear structure extending from the anal crypt to the fluid collection was detected. Fistula-in-ano with associated perianal abscess was diagnosed, and surgical incision with antibiotic therapy was performed.

**Figure 6:**
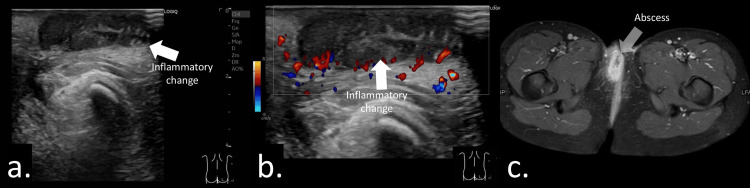
Inflammatory disease presenting with anal pain at the 11 o’clock position in a 12-year-old female. (a, b) Sagittal sonograms demonstrating a hypoechoic lesion surrounding a hypervascular area. No fluid collection was observed. (c) Axial contrast-enhanced MRI showing a small non-enhancing area surrounded by an enhancing region. The lesion measured 5 mm, and seton placement was not feasible due to its small size.

A perianal abscess typically appears as a hypoechoic mass surrounded by echogenic subcutaneous tissue, often with a hypervascular rim indicating inflammation [2,12,22] (Figures 5a, 5b; Video 2). Video 2 demonstrates an abscess within the dermis connected to a fistulous tract originating from the anal crypt. The fluid-filled cavity represents pus accumulation and generally warrants incision and drainage. Although most abscesses are readily detected on ultrasonography, some may be difficult to visualize because of atypical presentation or deep location [2]. Assessment of abscess size is important, as small abscesses may be insufficient to permit seton placement (Figures 6a-6c). Ultrasonography also provides real-time guidance and is useful for aspiration or puncture of the abscess.

**Video 2:** Video demonstrating perianal abscess and fistula-in-ano at the 11 o’clock position in a six-month-old boy. Sagittal view of the anal canal obtained with the probe parallel to the intergluteal cleft. A hypoechoic fistulous tract is visualized extending from the anal canal to the abscess. A diagnosis of fistula-in-ano with associated perianal abscess was made, and a surgical incision was performed.

Patients’ characteristics

Fistula-in-Ano and Perianal Abscess in Infants

Infantile fistula-in-ano and perianal abscesses are relatively common, occurring in approximately 4% of infants [3]. The condition may be related to abnormalities of the crypts of Morgagni and elevated androgen levels [3]. In this population, ultrasonography is particularly advantageous, as it can be performed rapidly at the bedside without exposure to radiation or the need for general anesthesia (Figures 3, 5; Video 2).

Fistula-in-Ano and Perianal Abscess in Children With Inflammatory Bowel Disease

**Figure 7:**
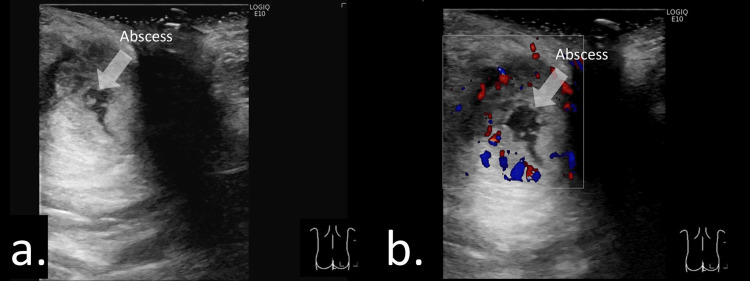
Inflammatory bowel disease presenting with an anal ulcer and pain at the 12 o’clock position in a 12-year-old female. (a, b) Sagittal sonograms demonstrating a small hypoechoic abscess measuring 9 mm, surrounded by reactive hypervascular changes.

Inflammatory diseases are sometimes accompanied by perianal abscesses and fistula-in-ano, which may be their initial manifestation [6,12,24]. Inflammation of the anal and rectal mucosa (e.g., proctitis) can predispose to fistula formation and is associated with a poorer prognosis [25]. Treatment planning often depends on the presence or absence of rectal inflammation [14,18]. Because transabdominal ultrasound provides limited visualization of the rectum, perianal ultrasonography is particularly useful for assessing these lesions [14,26]. Additionally, it can help evaluate the degree of inflammatory activity, correlating well with endoscopic findings [11,14,18]. Rectal wall thickening and hypervascularity are key indicators of active disease [11]. Figure 7 and Video 3 show the findings of inflammatory bowel disease with a small hypoechoic abscess surrounded by inflammatory changes.

**Video 3:** Video demonstrating inflammatory bowel disease presenting with an anal ulcer and pain at the 12 o’clock position in a 12-year-old female. Sagittal view of the anal canal obtained with the probe placed parallel to the intergluteal cleft. A small hypoechoic abscess surrounded by inflammatory changes is visualized.

Fistula-in-Ano and Perianal Abscess in Immunocompromised Children

**Figure 8:**
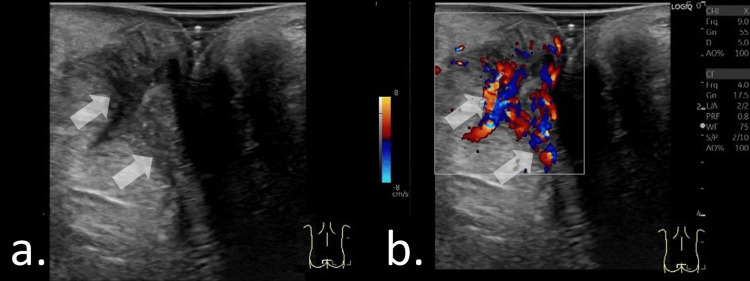
Chemotherapy for a right-sided anal pain and perianal inflammation without abscess formation in a 17-year-old female. (a, b) Coronal views of the anal canal obtained with the probe placed perpendicular to the gluteal cleft. Hypervascular inflammatory changes were visualized. No hypoechoic fluid collection was detected.

Managing fistula-in-ano and perianal abscesses in immunocompromised patients is challenging [20,27]. Delays in treatment may lead to bacteremia and sepsis and can be associated with increased mortality [23]. Therefore, rapid and accurate diagnosis is essential [22]. In pediatric patients with impaired immune responses, abscess formation and clinical signs may be subtle, making diagnosis based solely on physical examination difficult [28,29]. In such cases, ultrasonography provides critical diagnostic information to facilitate timely and appropriate management (Figure 8).

Imaging strategy for managing patients with fistula-in-ano and perianal abscess

The diagnostic performance of perianal ultrasonography is operator-dependent; therefore, we propose an imaging strategy for the management of patients with fistula-in-ano and perianal abscesses [30]. In patients with suspected perianal disease, bedside physical examination remains essential, followed by perianal ultrasonography. However, in cases involving deep-seated lesions or large abscesses, evaluation with ultrasonography alone may be insufficient. MRI is an essential modality for comprehensive assessment in such patients. Nevertheless, MRI is often costly and may require general anesthesia, particularly in pediatric populations. MRI should be considered when ultrasonographic evaluation is inconclusive or when complicated anal fistulas are suspected. Additionally, perianal ultrasonography is valuable for monitoring response to treatment. This proposed imaging strategy may help guide further diagnostic evaluation and inform appropriate treatment selection.

Discussion

Perianal ultrasonography is not a familiar technique for many sonographers. Nevertheless, it is a valuable method for visualizing fistula-in-ano and perianal abscesses and can provide important information for patient management.

Fistula-in-ano and perianal abscess are among the most common anorectal conditions in pediatric patients [1,22]. Initial management may include antibiotic therapy and abscess incision and drainage [3]. In some cases, particularly those associated with underlying disease, urgent or disease-specific treatment may be required [20,23,25,31]. Therefore, accurate diagnosis is essential to guide appropriate management. Perianal ultrasonography is particularly advantageous in pediatric patients because it does not involve radiation exposure or require general anesthesia. Developing proficiency in this technique is therefore important for sonographers.

Despite its usefulness, ultrasonography may not always visualize the entire lesion because of the complex anatomy of fistulous tracts or the large size of abscesses. In addition, compared with pediatric patients, adults are less likely to undergo perianal ultrasonography due to larger body habitus and technical limitations. In such cases, MRI is helpful for delineating the extent and structure of perianal abscesses and fistula-in-ano. Diffusion-weighted imaging and gadolinium-enhanced T1-weighted imaging can usually depict the full extent of the lesion clearly [8,10]. However, MRI is more costly than ultrasonography, and concerns regarding gadolinium deposition have been raised; therefore, ultrasonography remains useful as an initial modality and for selecting patients who require further MRI evaluation [32,33].

With ultrasonography, it may be difficult to distinguish between an open fistula and a linear hypoechoic scar. Seton therapy is performed in cases with open fistulas; thus, it is not indicated in cases without an active tract, and ultrasonography alone may not reliably differentiate these conditions [3,20,31]. This represents a limitation of the modality, and clinical findings, particularly indicators of disease activity, are important to support imaging interpretation.

Although the perianal ultrasonographic approach is useful, adequate pain control is often necessary [2]. Anal pain is the most common presenting symptom in patients with fistula-in-ano and perianal abscess, whereas asymptomatic cases are uncommon [7,22]. The probe is typically placed over the area of maximal tenderness, and repeated examinations may be required to monitor lesion size and progression. Therefore, providing a clear explanation of the procedure to patients and their families is important, and in some cases, administration of analgesics before the examination should be considered.

Take-home point

Perianal ultrasonography is performed as follows: the patient is placed in the lateral decubitus or prone position. To obtain a sagittal view, the probe is positioned on the proctodeum parallel to the intergluteal cleft to acquire a longitudinal view of the anus and rectum. The probe is then gently swung from right to left to evaluate the surrounding structures. The locations of fistulas and abscesses are identified based on anatomical landmarks and the anal clock orientation. Fistula-in-ano is visualized as a hypoechoic tract extending from the rectal crypt to the dermis, whereas a perianal abscess appears as a hypoechoic mass surrounded by echogenic subcutaneous tissue. Surgical incision or drainage is typically selected based on these imaging findings. However, the diagnostic performance of perianal ultrasonography is operator-dependent. In cases involving deep-seated lesions or large abscesses, evaluation with ultrasonography alone may be insufficient. In such situations, MRI serves as the reference standard for comprehensive assessment.

## Conclusions

Perianal ultrasonography is a valuable imaging modality for evaluating perianal lesions, and sonographers should become familiar with this technique. A sagittal view of the anus and rectum is particularly useful for identifying the anatomical structures of the anal canal and determining the location of fistulas and abscesses while gently swinging the probe. This approach provides important information for the management of pediatric patients with fistula-in-ano and perianal abscess, helping guide treatment selection, such as surgical incision or antibiotic therapy. However, careful patient management, especially adequate pain control, is essential during each examination. In selected cases, additional evaluation with MRI should also be considered.
